# Efficacy and safety of D-TACE followed by D-RFA for unresectable large hepatocellular carcinoma

**DOI:** 10.3389/fonc.2025.1530951

**Published:** 2025-07-28

**Authors:** Qingqing Pang, Wenping Luo, Shaojun Chen, Hua Zhou, Riguang Zhang, Yueyong Li, Jianbo Zhao, Chunwang Yuan, Guodong Wang

**Affiliations:** ^1^ Department of Oncology, the Fourth Affiliated Hospital of Guangxi Medical University, Liuzhou, Guangxi, China; ^2^ Department of Blood Transfusion, the Fourth Affiliated Hospital of Guangxi Medical University, Liuzhou, Guangxi, China; ^3^ Department of Oncology, Changsha Central Hospital, University of South China, Changsha, China; ^4^ Division of Vascular and Interventional Radiology, Department of General Surgery, Nanfang Hospital, Southern Medical University, Guangzhou, Guangdong, China; ^5^ Liver Disease and Cancer Interventional Therapy Center, Beijing Youan Hospital, Capital Medical University, Beijing, China

**Keywords:** drug-loaded microsphere transarterial chemoembolization, double-needle radiofrequency ablation, unresectable, large, hepatocellular carcinoma

## Abstract

**Objective:**

To evaluate the safety and short-term clinical efficacy of drug-loaded polyvinyl alcohol microspheres transarterial chemoembolization (D-TACE) in the treatment of unresectable large liver carcinoma with sequential double-needle water-cooled circulating radiofrequency ablation (D-RFA).

**Methods:**

A retrospective analysis was performed for patients with large hepatocellular carcinoma who underwent sequential D-TACE with D-RFA treatment at our hospital. From May 2019 to May 2023, a total of 143 intrahepatic malignant lesions were treated, and a total of 110 D-TACE and 96 D-RFA interventional therapy procedures were performed. The short-term efficacy at 1, 3, and 6 months after interventional therapy was analyzed based on the modified Response Evaluation Criteria in Solid Tumor (2020 edition) criteria. The evaluation included the efficacy of local tumor control, feasibility of technical implementation, safety of surgery, and tolerability by surgical patients.

**Results:**

Sixty-two patients underwent successful interventional therapy, achieving good technical feasibility. The objective response rate (ORR) at 3, 6, and 12 months was 90.4%, 85.5%, and 74.2%, respectively. The median overall survival (OS) was 35.0 months (95% CI: 24.7-45.3). The survival rates at 3, 6, and 12 months were 100% (62/62), 96.7% (60/62), and 93.5% (58/62), respectively. No cases of death occurred due to serious complications such as ectopic embolism, tumor rupture, or liver failure within 1 month after surgery. Two cases of postoperative tumor lysis syndrome, 3 cases of pleural effusion caused by intercostal artery injury, and 4 cases of small effusion in the abdominal cavity were reported. Eight cases of mild, moderate, and severe abdominal pain during or after the operation; 6 cases had mild to moderate liver function impairment. Eight patients experienced fever within 1 week after surgery.

**Conclusion:**

The D-TACE with sequential D-RFA technique for the treatment of unresectable large liver cancer is safe and controllable, with a high ORR. This combination treatment provides a useful reference value for the exploration of new treatment modes for advanced liver cancer, but the long-term efficacy evaluation requires multi-center, large-sample clinical studies, and continuous follow-up data analysis.

## 1 Introduction

Primary liver cancer is the sixth most common cancer and the third leading cause of cancer-related mortality worldwide. In 2020, approximately 906,000 new cases and 830,000 deaths were reported. Liver cancer ranks fifth in global incidence, with significantly higher incidence and mortality rates among men than women, and ranks as the second leading cause of cancer death among male populations (1). In China, liver cancer accounts for about 40% of global cases and deaths. Although the incidence has declined compared to two decades ago, the mortality rate remains the second highest, presenting a continuing public health challenge. Hepatocellular carcinoma (HCC) accounts for roughly 75% of primary liver cancer cases. Due to its nonspecific early-stage symptoms, high malignancy, and rapid progression, HCC is frequently diagnosed at advanced stages, contributing to poor clinical outcomes (2, 3). Only 20% to 30% of patients are eligible for surgical resection, and the 5-year recurrence rate after surgery is as high as 50% to 70% (4). In recent years, treatment modalities for advanced liver cancer, including transarterial chemoembolization (TACE), thermal ablation, systemic therapies, and radiotherapy, have been increasingly utilized, demonstrating improvements in objective response rates and quality of life (5–7). Despite therapeutic advances, local tumor progression persists as a major clinical challenge in monotherapy regimens, underscoring the necessity for multimodal and personalized treatment strategies to optimize patient outcomes and mitigate disease progression risks (8, 9). TACE is primarily used for treating HCC that is inoperable or unsuitable for surgical intervention. Nevertheless, TACE monotherapy exhibits suboptimal tumor necrosis rates and is frequently complicated by tumor recurrence due to incomplete eradication of residual lesions (10). Conversely, ablation techniques have demonstrated the potential to be effective, not only for tumors <3 cm, but also for tumors >5 cm (11, 12). Notably, the combined application of TACE and ablation therapy yields synergistic effects, significantly enhancing tumor necrosis while improving overall survival rates compared with single-modality approaches (13, 14).

Large HCC presents unique challenges due to high tumor burden, insufficient residual liver volume, poor liver reserve function, vascular invasion, and early intrahepatic metastases. These features collectively contribute to low surgical resectability rates, unfavorable prognosis, and significantly impaired quality of life (15). These challenges make it a major public health problem requiring urgent solutions. Contemporary clinical practice, multidisciplinary and multi-modal treatment approaches for advanced liver cancer are widely recognized and recommended in various clinical guidelines, including the Chinese Medical Association Guidelines for Liver Cancer, the American Association for the Study of Liver Diseases (AASLD) Guidelines, and the European Association for the Study of the Liver (EASL) Guidelines (4, 16). The 2024 Chinese Guidelines for the Diagnosis and Treatment of Primary Liver Cancer recommend that TACE combined with ablation offers better outcomes than TACE alone for tumors >5 cm. Based on this recommendation, our study specifically targeted patients with large HCC, defined as having at least one intrahepatic lesion ≥5 cm in diameter (17) and massive HCC tumors were defined as being at least 10 cm in diameter (18). The study protocol incorporated two significant technical innovations to enhance conventional TACE plus radiofrequency ablation (RFA) therapy: drug-eluting bead TACE (D-TACE) and dual-electrode RFA (D-RFA) for synchronous multipolar ablation. Following comprehensive post-interventional assessment, enrolled patients received 2 to 4 treatment cycles administered in a staged therapeutic approach. This study summarizes the outcomes of 62 patients with unresectable large HCC who underwent D-TACE followed by D-RFA. Through a comprehensive analysis of the data, the study aimed to assess the short-term efficacy, safety, and clinical applicability of this approach.

## 2 Materials and methods

### 2.1 Patient information

This retrospective cohort study evaluated 62 consecutive patients with large HCC who underwent D-TACE followed by D-RFA at our tertiary medical center between May 2019 and May 2023. The cohort comprised 47 male (75.8%) and 15 female (24.2%) patients, with a cumulative total of 143 intrahepatic lesions treated. The inclusion criteria for patient selection were as follows: patients diagnosed with HCC based on pathological confirmation via biopsy or meeting the clinical diagnostic criteria for liver cancer (19); presence of ≥1 measurable intrahepatic lesion meeting either a single tumor diameter≥5 cm, or multiple lesions with a combined diameter >5 cm; Eastern Cooperative Oncology Group (ECOG) performance status score ≤2; Child-Pugh liver function class A or B (20); ineligible for surgical resection based on multidisciplinary consultation or refusal of surgical resection by the patient. The exclusion criteria were age <18 years or >85 years; ECOG score ≥3; Barcelona Clinic Liver Cancer (BCLC) Stage D or Chinese Liver Cancer (CNLC) Stage IV; estimated survival <3 months, or the presence of severe underlying diseases, bleeding tendency, severe infections, organ failure (heart, lung, liver, or kidney), refractory ascites, or type IV portal vein tumor thrombus (VP4); extensive tumor burden (>6 intrahepatic lesions or total tumor diameter >20 cm); patients with psychiatric disorders affecting self-care or inability to lie in the required surgical positions (prone or supine) during the procedure.

This study was approved by the Ethics Committee of Liuzhou Workers’ Hospital (KY2022008). All patients or their family members were fully informed about the interventional therapy plan, associated risks, costs, and prognosis. Informed consent forms and other relevant documents were signed by all participants.

### 2.2 Equipment and materials

Angiographic catheters and vascular sheaths size 5F (Terumo Corporation, Japan); microcatheters size 2.7F (Boston Scientific, USA), callispheres drug-loaded polyvinyl alcohol (PVA) embolic microspheres (100–300 μm, 1 g; Jiangsu Hengrui Medicine Co., China); RFA System, model AJ-500A (2 units; Anjun Medical Technology Co., Nanjing, China); disposable water-cooled circulating RF electrodes, model RFE-3SN (17G150–30 compatible with the AJ-500A system); digital subtraction angiography (DSA) instrument (Philips); CT simulation localization system model SOMATOM Definition AS (Siemens, Germany).

### 2.3 Interventional therapy procedure

#### 2.3.1 D-TACE procedure

The D-TACE procedure followed the standard protocols of disinfection, draping, and vital sign monitoring. The steps involved were as follows: (i) preoperative analgesia: 15 minutes before the procedure, 1 mg of hydromorphone (Yichang Humanwell Pharmaceutical Co.) was administered intravenously at a slow rate for pain relief; (ii) vascular access and angiography was performed using the modified Seldinger technique. The femoral artery was percutaneously punctured under local anesthesia and a 5F vascular sheath was inserted. A 5F angiographic catheter was guided into position with a hydrophilic guidewire to perform hepatic and superior mesenteric artery angiography; (iii) angiographic analysis was performed to identify the location, size, number of tumors, and the distribution of feeding arteries. For some patients, additional angiography through the right phrenic artery or internal thoracic artery was required to supplement the imaging; (iv) for super-selective catheterization and chemotherapy injection: a 2.7F microcatheter was used to perform super-selective catheterization of the main arterial branches supplying the tumor. The location was confirmed by angiography. Lobaplatin (30 mg/m²) dissolved in 50 mL was slowly injected in 5% glucose solution over 15–20 min; (v) for embolization with Drug-loaded microspheres, epirubicin (25 mg/m²) was dissolved in 5 mL of 5% glucose solution and mixed with blue drug-loaded microspheres (100–300 µm). The solution was allowed to stand for 10–15 minutes to ensure proper drug loading. The loaded red microspheres were mixed with 10–15 mL of contrast agent and the mixture was slowly injected into the tumor’s arterial supply to perform precise embolization. Blank microspheres of different sizes were used to supplement embolization based on intraoperative DSA results. The embolization endpoint was reached when ≥90% of the tumor vessels disappeared on DSA or based on tumor characteristics and patient tolerance; and (v) perioperative management and postoperative care involved administering antiemetics, antiallergics, analgesics, and liver-protective medications during the perioperative period. After the procedure, compression bandages were applied for 6 h and blood pressure, pulse, and dorsal pedis artery pulse were monitored. Symptomatic treatment was provided as needed for the patient’s recovery.

#### 2.3.2 D-RFA treatment

The D-RFA procedure is performed sequentially 1–4 weeks after D-TACE, following the treatment plan. The perioperative preparation included preoperative analgesia given 15 minutes before the procedure, for which 1 mg of hydromorphone was administered slowly via intravenous injection to relieve pain. Electrode placement was performed using two sets of electrode pads attached to the outer sides of both thighs, with wires connected to the ablation devices. The patient was positioned, and enhanced computed tomography (CT) imaging of the upper abdomen was performed for localization. The contrast agent used was iodixanol (Jiangsu Hengrui Pharmaceuticals Co., Ltd.) infused at 50–70 mL at 3.0 mL/s. Image acquisition included arterial and portal venous phases. For pre-ablation quality control, the puncture path, needle insertion angle, type of ablation needle, and number of needles are determined based on the imaging results. CT guidance were set as follows: voltage, 120 kV; current, 80 mA. The scanning range was ±50 mm from the target puncture point along the head-to-tail axis. The patient was disinfected, draped, provided with oxygen, and monitored continuously using a patient monitor.

#### 2.3.3 Intraoperative procedure

Needle insertion was performed after administering local anesthesia with lidocaine, the operator performed the puncture and needle placement manually under non-real-time CT guidance, following the pre-planned path. Needle placement confirmation and ablation relied on CT images to confirm that both ablation needles had reached the target. Each needle was connected to a separate RFA device, which was activated simultaneously. The settings were as follows: power, 80–120 W; duration, 15 min; target temperature, 85–95°C; needle spacing, 2–3 cm; organ protection when necessary, was performed by inserting a 5F side-hole drainage catheter percutaneously to infuse cold saline, to protect nearby organs from thermal injury.

The adjusting needle positions were adjusted under CT guidance to ensure complete tumor coverage. Ablation cycles and times were adjusted according to the preoperative plan. The needle track ablation and final evaluation were performed by slowly withdrawing the needles while performing track ablation to prevent bleeding. An enhanced CT scan was conducted to evaluate the ablation outcome and check for complications.

For postoperative care, if no significant bleeding or severe complications were found, the patient was transferred to the ward. Vital signs were monitored for 24 hours, and antibiotics were administered prophylactically to prevent infection.

### 2.4 Systemic treatment

Sorafenib, donafenib, or lenvatinib tyrosine kinase inhibitors (TKIs) were administered as follows: sorafenib as an oral administration of 400 mg twice daily, oral donafenib (200 mg) was administered twice a day, and lenvatinib as an oral administration of 8 mg (for patients weighing <60 kg) or 12 mg (for those weighing >60 kg) once daily, following practice guidelines and expert consensus. TKIs were discontinued for 3 days before and after the interventional treatment session. The immune checkpoint inhibitors (ICIs) camrelizumab, tislelizumab, and sintilimab were intravenously administered at a dose of 200 mg every 3 weeks.

### 2.5 Follow-up

All enrolled patients underwent monthly contrast-enhanced CT or magnetic resonance imaging (MRI) scans. Laboratory assessments included serum alpha-fetoprotein (AFP) levels and comprehensive liver function evaluation (Child-Pugh score). The primary endpoint was OS, and the secondary endpoints were tumor response, ORR, and disease control rate (DCR). OS was defined as the duration from initial treatment to death from any cause or last follow-up. The mRECIST 2020 criteria were used to evaluate short-term therapeutic efficacy (21). The response was divided into complete response (CR), partial response (PR), stable disease (SD), or progressive disease (PD) depending on the results. The ORR included both CR and PR, whereas the disease control rate (DCR) was calculated as the combination of CR, PR, and SD. The treatment strategy during follow-up considered evidence of tumor residue, for which the patient proceeded to the next cycle of D-TACE followed by D-RFA. If tumor progression was observed, the case was referred to multidisciplinary tumor board (MDT) for alternative treatment strategy formulation. All 62 cases were followed up until May 2024. The complications were documented according to the Society of Interventional Radiology (SIR) Clinical Practice Guidelines classification system.

### 2.6 Data collection and statistical analysis

This retrospective, single-center study focused on the treatment outcomes of D-TACE followed by D-RFA for unresectable large HCC. The data collected included age, sex, stage, Child-Pugh class, tumor size, tumor number, number of treated lesions, change of AFP levels and liver function, types and incidence of severe postoperative complications, number of D-TACE and combined ablation procedures performed. For follow-up evaluations, patients underwent systematic follow-ups and objective assessments 4 to 6 weeks after interventional therapy. Enhanced MRI or MRI with gadobenate dimeglumine (Gd-BOPTA) was recommended for imaging evaluation. Imaging results were independently assessed by two senior physicians, including one senior radiologist specializing in diagnostic imaging.

Data were processed using SPSS v.22.0 statistical software. Normally distributed data are expressed as mean ± standard deviation (x¯ ± s). T-tests were used for continuous variables. Chi-square tests were applied for categorical variables. Survival analyses were performed using the Kaplan–Meier method including relevant variables univariate analysis. The Cox proportional hazards model was used to perform multivariate survival analysis, assessing the impact of factors such as age, sex, presence of tumor thrombus, liver function grade, degree of ascites, ascites, HBsAg status, Chinese Liver Cancer (CNLC) stage, Barcelona Clinic Liver Cancer (BCLC) stage, Child-Pugh class, tumor number, and AFP levels on survival outcomes. Cases were presented as numbers/percentages (n/%). A P-value < 0.05 was considered statistically significant.

## 3 Results

### 3.1 Patient characteristics

**Figure 1:**
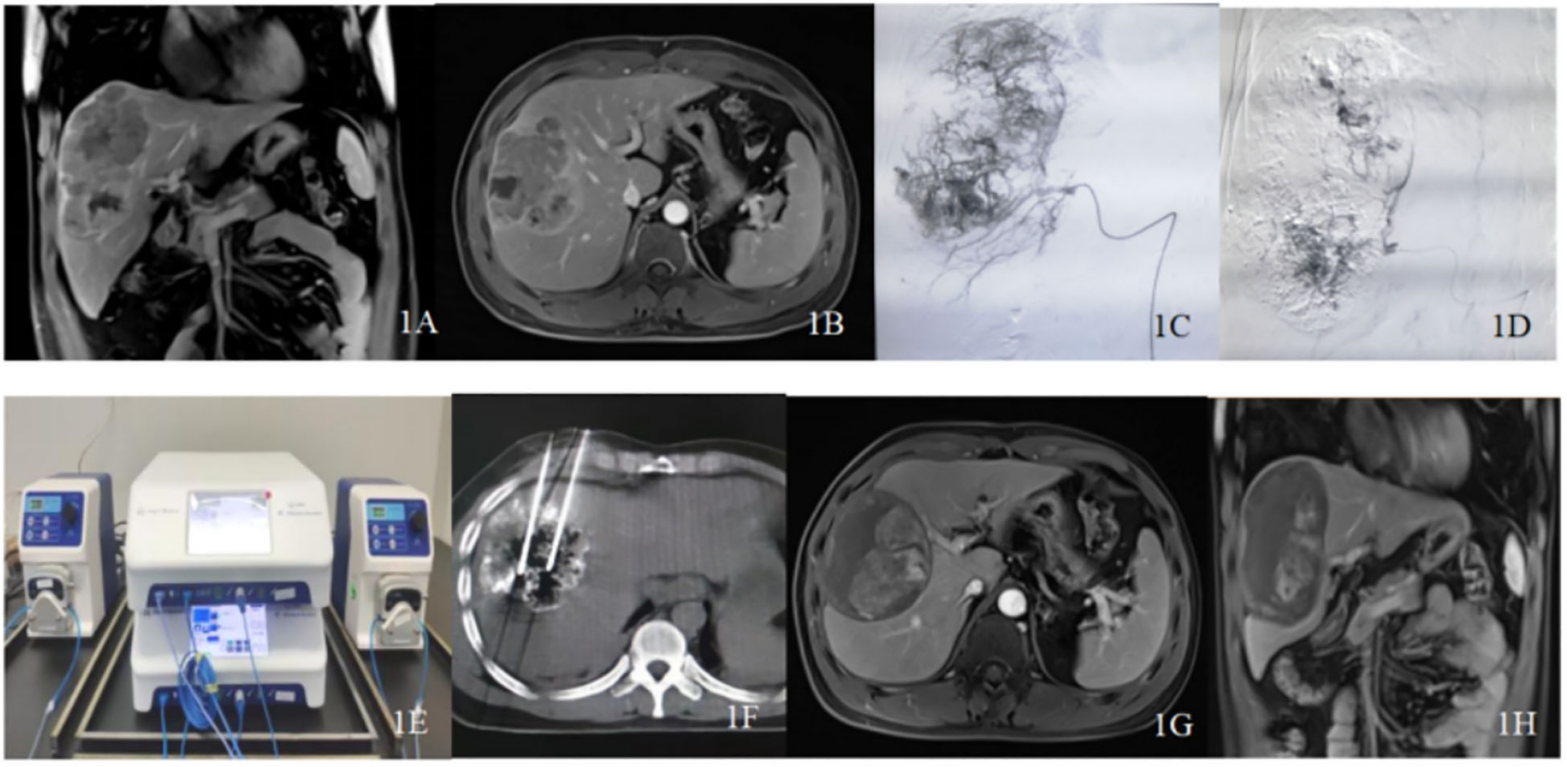
Typical case patient details: A 37-year-old male diagnosed with primary liver cancer (massive type) and multiple intrahepatic metastases underwent drug-loaded polyvinyl alcohol microspheres transarterial chemoembolization (D-TACE) followed by sequential double-needle water-cooled circulating radiofrequency ablation (D-RFA) treatment. His alpha-fetoprotein (AFP) levels decreased from >2000 ng/ml to normal. **(A, B)** Abdominal magnetic resonance image (MRI) enhancement indicates a large tumor in the right liver lobe with multiple sub-nodules, with a maximum diameter of approximately 12.0 cm and remaining liver volume <50%. **(C, D)** Intraoperative angiography during D-TACE shows a large tumor nest, with significant reduction in tumor staining post-operation. **(E)** Dual radiofrequency devices and dual water-cooled circulation pumps working in tandem. **(F)** computed tomography (CT)-guided dual radiofrequency electrodes used to ablate the liver tumor. **(G, H)** Twelve months post-intervention follow-up with abdominal MRO shows no enhancement of the tumor. This comprehensive imaging illustrates the effectiveness of the treatment and the patient’s progress over time.

A total of 62 patients were included in this study, the baseline characteristics of patients with large HCC are summarized in **Table 1**. Most patients were male (75.8%), and the median age was 52.53 ± 11.54 years. In total, 50 patients had ECOG 1 and 52 were Child-Pugh A. The number cases with tumor diameters ≤7 cm was 27; 14 cases had tumors 7–10 cm, and tumors ≥10 cm were found in 21 cases. The average tumor diameter was 8.62 ± 4.48 cm and the average number of intrahepatic lesions treated were 2.30 per patient; Fifteen patients had extrahepatic metastasis, and 29 had portal vein tumor thrombus. Over 50% of patients were BCLC C the majority of patients (80.6%) were hepatitis B virus surface antigen (HBsAg)-positive. Fifty-five patients received synchronous treatments, including TKIs monotherapy (n = 26), ICIs (n = 2), and targeted therapy combined with immunotherapy (n = 27). Decompensated liver cirrhosis was observed in 10 cases. Overall, D-TACE sessions involved 110 procedures, D-RFA sessions included 96 procedures, and the number of ablated lesions was 143. Representative cases are presented in **Figure 1**.

**Table 1:** Patient demographics and baseline characteristics (n = 62).

Characteristics	Variable	All patients (n=62)	Percentage %
Sex	Male	47	75.8
	Female	15	24.2
Age (years)	≤60 >60	50 12	80.6 19.4
Age, years, mean			52.53 ± 11.54
ECOG Score	0	11	17.8
	1 2	50 1	80.6 1.6
CNLC stage	I II III	7 12 43	11.3 19.4 69.4
BCLC stage	A	12	19.4
	B C	16 34	25.8 54.8
Child-Pugh class	A	52	83.9
	B	10	16.1
AFP (ng/mL)	<400	40	64.5
	≥400	22	35.5
Tumor number	1-2	41	66.1
	≥3	21	33.9
Tumor size (cm)	5-≤7	27	43.5
	7-10	14	22.6
	≥10	21	33.9
Tumor size, mean (cm)			8.62 ± 4.48
Extrahepatic metastasis	Absent	47	75.8
	Present	15	24.2
Portal vein tumor thrombus	Absent	33	53.2
	Present	29	46.8
HBsAg	positive	50	80.6
	negative	12	19.4
Systemic Treatment	TKIs	26	41.9
	ICIs	2	3.3
	TKIs+ICIs	27	43.5
	None	7	11.3

ECOG, Eastern Cooperative Oncology Group; CNLC, China liver cancer staging; BCLC, Barcelona Clinic Liver Cancer; AFP, alpha-fetoprotein; HBsAg, hepatitis B virus surface antigen; TKIs, tyrosine kinase inhibitors; ICIs, immune checkpoint inhibitors.

### 3.2 Survival analysis

**Figure 2:**
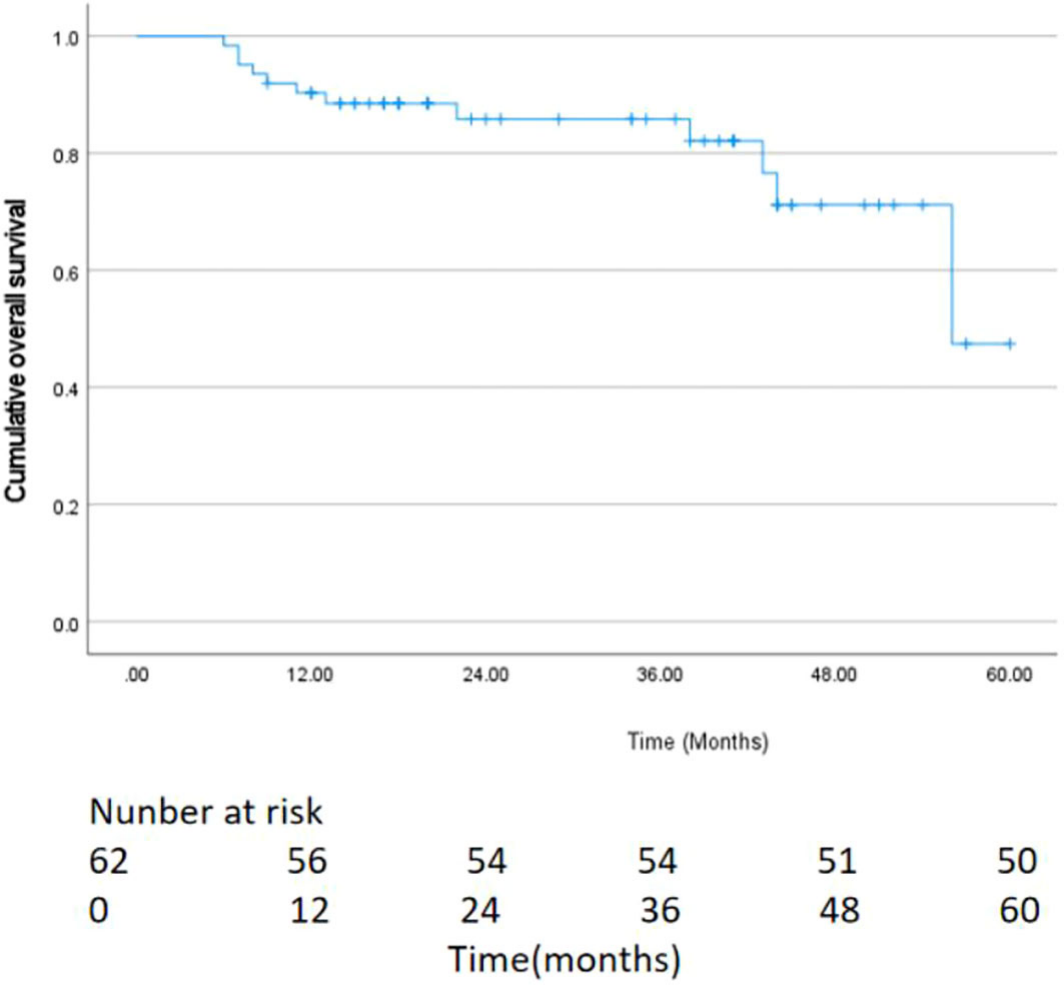
Kaplan–Meier curves of overall survival (OS).

As of May 2024, 62 patients were followed up, with follow-up durations ranging from 6 to 60 months (average 28.47 ± 15.46 months), and a median follow-up time of 23.5 months, with no patient lost to follow-up. The median OS was 35.0 months (95% CI:24.7–45.3) (**Figure 2**). Postoperative survival rates at 3, 6, and 12 months were 100% (62/62), 96.7% (60/62), and 93.5% (58/62), respectively. The best response evaluation results are shown in **Table 2**. According to the mRECIST criteria, at 3 months post-evaluation 5/51/5 patients had the best tumor response of CR/PR/SD, and one case was PD 1.6% (1/62). A CR was observed in 12 patients (19.4%), a PR in 41 patients (66.1%), SD in 3 patients (4.8%), and PD was in 6 patients (9.7%) at 6 months. At 12 months post-evaluation, 20 patients (32.3%) achieved CR, PR was achieved in 26 patients (41.9%), whereas SD was 6.5% (4/62) and PD was 9.7% (19.4%/62). The ORR at 3, 6, and 12 months post-combined therapy were 90.4%, 85.5%, and 74.2%, respectively. The DCR were 98.5%, 90.3%, and 80.7% at the same intervals.

**Table 2:** Patient response according to mRECIST outcomes (n = 62).

Efficacy	3 months post-evaluation	6 months post-evaluation	12 months post-evaluation
CR	5 (8.1%)	12 (19.4%)	20 (32.3%)
PR	51 (82.3%)	41 (66.1%)	26 (41.9%)
SD	5 (8.1%)	3 (4.8%)	4 (6.5%)
PD	1 (1.6%)	6 (9.7%)	12 (19.4%)
ORR (CR+PR)	56 (90.4%)	53 (85.5%)	46 (74.2%)
DCR (CR+PR+SD)	61 (98.5%)	56 (90.3%)	50 (80.7%)

mRECIST, modified Response Evaluation Criteria in Solid Tumors; CR, complete response; PR, partial response; SD, stable disease; ORR, objective response rate; DCR, disease control rate.

### 3.3 Safety analysis

All 62 patients underwent at least one session of D-TACE followed by sequential D-RFA, with a 100% procedural success rate. A total of 110 D-TACE sessions were performed, targeting 143 lesions, followed by 96 sessions of D-RFA. The incidence of moderate and severe adverse reactions or complications was 16.1% (10/62) and 1.6% (1/62), respectively. Tumor lysis syndrome occurred in two patients (1 mild, 1 moderate). Ascites was observed in 4 cases (3 mild, 1 moderate). Abdominal pain occurred in 7 patients (5 with mild and 2 moderate) that arose during ablation. Transient liver function impairment was reported in 6 patients (4 mild, 2 moderate), mostly appearing one-week post-TACE. Thrombocytopenia occurred in 2 cases. Intercostal artery injury was reported in 3 cases, with 1 patient developing moderate pleural effusion postoperatively and another with large pleural effusion, both resolving after symptomatic management. The most common adverse effects associated with D-TACE followed by D-RFA included mild fever, vomiting, and loss of appetite. No severe postoperative complications such as large hepatic tumor rupture, ectopic embolization, gastrointestinal perforation, or liver or renal failure were observed (**Table 3**).

**Table 3:** Summary of common complications (n = 62).

Adverse events	Mild	Moderate	Severe
Tumor lysis syndrome	1	1	0
Ascites	3	1	0
Abdominal pain	5	2	0
Transient liver function impairment	4	2	0
Intercostal artery injury	1	1	1

### 3.4 Liver function analysis and AFP changes

Liver function indicators were measured at baseline (pre-therapy) and at one-week post-intervention. Alanine aminotransferase (ALT) levels were 60.58 ± 49.99 U/L, alkaline phosphatase (ALP) 133.48 ± 80.04 U/L, aspartate aminotransferase (AST) 74.61 ± 77.08 U/L, and total bilirubin (TBIL) 20.10 ± 17.99 μmol/L. At one week post-treatment, ALT decreased to 42.44 ± 42.88 U/L, ALP increased to 163.15 ± 13.05 U/L, AST was 69.66 ± 97.26 U/L, and TBIL was 18.27 ± 12.85 μmol/L. Significant differences were observed in ALT (P=0.025) and ALP (P=0.040) between pre- and post-treatment values. In contrast, AST and TBIL showed no significant changes (P>0.05), suggesting stable bilirubin metabolism (**Table 4**). Preoperative AFP levels were stratified as follows: 35.5% (22/62) of patients exhibited AFP levels ≥400 ng/mL. The mean preoperative AFP level was 22,619.7 ± 55,686.47 ng/mL, showing no significant change (26,588.18 ± 116,201.47 ng/mL; P>0.05) at 1-month post-treatment.

**Table 4:** The changes in serum AFP and liver function indicators pretherapy and post-treatment.

Indicator	Preoperative	Postoperative	P Value
ALT (U/L)	60.58 ± 49.99	42.44 ± 42.88	0.025
ALP (U/L)	133.48 ± 80.04	163.15 ± 13.05	0.040
AST (U/L)	74.61 ± 77.08	69.66 ± 97.26	0.589
TBIL (μmol/L)	20.10 ± 17.99	18.27 ± 12.85	0.446
AFP`(x ± s,ng/mL)	22619.72 ± 55686.47	26588.18 ± 116201.47	0.786

ALT, alanine transaminase; ALP, alkaline phosphatase; AST, aspartate transaminase; TBIL, total bilirubin; AFP, alpha-fetoprotein.

### 3.5 Analysis of prognostic factors

The univariate analysis indicated that ascites and post-AFP were significant risk factors. In the multivariate analysis, post AFP ≥400 ng/mL, were independent predictors of OS (**Table 5**).

**Table 5:** Univariate and multivariate analysis of factors related to OS.

Characteristics	Univariate analysis				Multivariate analysis			
	HR	95%CI		P Value	HR	95%CI		P Value
Age, <45 vs. 45–60 vs. >60	0.875	0.374	2.048	0.759				
Portal vein tumor thrombus, absent vs. present	1.158	0.369	3.636	0.801				
Ascites, absent vs. present	3.268	0.895	10.736	**0.048**	2.930	0.887	9.678	0.078
Number of TACE, <3 vs. ≥3	0.354	0.045	2.762	0.322				
Number of ablation procedures, <3 vs. ≥3	1.212	0.260	5.636	0.807				
HBsAg, negative vs. positive	0.731	0.196	2.725	0.640				
CNLC stage, I-II vs. III	1.707	0.449	6.489	0.432				
BCLC stage, A vs. B vs. C	1.019	0.531	0.361	1.161				
Child-Pugh class, A vs. B	1.065	0.228	4.974	0.936				
Tumor size, <7 vs. 7–10 vs. >10–15 vs. >15	1.349	0.813	2.239	0.246				
Pre AFP, <400 vs. ≥400 ng/ml	2.558	0.684	9.565	0.163				
Post AFP, <400 vs. ≥400 ng/ml	4.259	1.149	15.778	**0.030**	3.949	1.061	14.699	**0.041**
AFP changes, decrease vs. increase	3.165	0.905	11.071	0.071				
Systemic Treatment TKIs vs. ICIs vs. TKI+ICIs vs. None	1.125	0.650	1.947	0.673				

HR hazard ratio; CI confidence intervals; TACE, transarterial chemoembolization; HBsAg, hepatitis B virus surface antigen; CNLC, Chinese Liver Cancer; BCLC, Barcelona Clinic Liver Cancer; AFP, alpha-fetoprotein; TKIs, tyrosine kinase inhibitors; ICIs, immune checkpoint inhibitors.Univariate analysis showed that ascites and elevated postoperative alpha-fetoprotein levels were significant (p < 0.05). Multivariate analysis revealed that postoperative alpha-fetoprotein levels ≥ 400 nanograms per milliliter were significant (p < 0.05).

## 4 Discussion

TACE and local tumor ablation represent two widely employed therapeutic approaches for HCC. Compared to surgical resection, these minimally invasive techniques are associated with reduced trauma, accelerated recovery, and better overall tolerability in most patients. However, traditional TACE has been regarded as a non-curative treatment option. This limitation stems from several factors. For instance, low tumor necrosis rates, which have potential for tumor proliferation, and the necessity for multiple treatments, all of which may contribute to progressive liver function impairment (22, 23). As a relatively new drug-delivering device, D-TACE demonstrates superior local tumor control and reduced systemic toxicity compared to conventional TACE (24). However, developing safe and effective treatment strategies for large hepatic tumors continues to pose significant clinical challenges, primarily due to chemotherapeutic dose limitations, substantial costs associated with embolic materials and psychological distress from repeated interventions. Percutaneous ablation is generally perceived as an effective treatment modality (25). A single RFA electrode can induce complete tumor necrosis within a 2 to 3 cm diameter range. By utilizing a single radiofrequency ablation device to drive dual electrode needles asynchronously, the effective ablation range can approach 4 cm while maintaining good safety and patient tolerance. For HCC lesions ≤3 cm, RFA monotherapy has demonstrated satisfactory oncological outcomes (26, 27). However, for larger lesions (>3 cm), RFA as a standalone therapy is generally not recommended. Current evidence supports combined therapy using TACE and RFA (28–31). This combination has demonstrated favorable therapeutic efficacy and acceptable safety profiles. Accumulating clinical evidence suggests that TACE-RFA combination therapy may reduce tumor progression rates and enhance survival outcomes, although its optimal efficacy continues to be investigated (32–35). A time-to-event meta-analysis included 21 studies involving 3413 patients. The study found that TACE combined with RFA was associated with better OS than TACE alone, as well as longer OS compared with RFA alone. Subgroup analyses by tumor size showed that for patients with a tumor >3 cm, the combined treatment also achieved a better outcome than RFA alone (36). A control group was not included because the inferior outcomes of single-modality therapies in this population have been well-documented. Previous studies included patients with small (~3-5cm), single tumors, whereas few studies have compared combination treatment for intermediate-stage or advanced disease (37). We focused on extending this evidence to larger tumors. Our study included tumor sizes >5 cm and found that combined treatment was effective, which may have relied on RFA reduction of overall tumor-cell resistance and improved efficacy of chemotherapy delivered by D-TACE, allowing higher concentrations of chemotherapeutic agents to accumulate near the tumor vascular bed (38). Therefore, combined use has significant advantages. Systemic therapies derived from recommendations from guidelines. A recent review summarized that locoregional therapies combined with systemic therapies achieve encouraging results for the management of HCC (39). Nearly 90% of patients in our study used systemic therapies, but the regimen different substantially. A standardized combined treatment study design is needed to verify the efficacy.

In our study, D-TACE followed by D-RFA sequential treatment for unresectable large HCC resulted in a 12-month ORR of 74.2%. Concurrently, 32.3% of patients achieved CR. Notably, the BCLC stage C patients accounted for a substantial proportion of the study population, and portal vein tumor thrombosis (PVTT) appeared to significantly influence median survival outcomes. The efficacy revealed that the number of patients achieving CR significantly increased over time, indicating that the long-term effects of treatment gradually manifested, with an increasing proportion of patients reaching CR by 12 months, highlighting the treatment’s effectiveness. The proportion of patients achieving PR reached its peak at the 3-month follow-up (82.3%), followed by a progressive decline that likely reflected disease progression in non-responders. This underscores the need for continuous monitoring and potential follow-up treatments. The number of patients with stable lesions decreased after 3 months, suggesting dynamic tumor response patterns where most lesions either responded or progressed, with only a minority maintaining long-term stability. The number of patients with disease progression significantly increased at 6 and 12 months, suggesting that some patients may not have responded well to treatment, with the risk of disease deterioration rising over time. However, the observed decline in PR rates and rising PD incidence (from 1.6% to 19.4%) underscore the biological heterogeneity of large HCC and the urgent need for personalized therapeutic algorithms with extended surveillance. Current evidence predominantly evaluates long-term outcomes through 5-year OS and progression-free survival (PFS) endpoints. As evidenced by Zhang et al., a 5-year survival rate of 52.0% for patients receiving TACE and RFA combined, compared with 43.2% for those undergoing RFA alone (38). A retrospective study reported the ORR was 76.2%, and the OS was 24 months in unresectable hepatocellular carcinoma (uHCC) patients who underwent D-TACE combined with lenvatinib and camrelizumab (40). In our study, we achieved a better survival with combination D-TACE and RFA therapy. Similarly, Endo et al. found that TACE-RFA significantly improved the 5-year OS for advanced HCC patients (60.4% vs. 22.8%, p = 0.045) (41). Various drugs have been loaded in D-TACE. Bi et al. reported the outcomes of D-TACE loaded with raltitrexed in patients with unresectable or recurrent HCC. The ORR and DCR at 1, 3, and 6 months after D-TACE were 72.0% and 96.0%, 57.1% and 85.7%, 47.6% and 66.7%, respectively. However, the use of raltitrexed may be limited by higher costs and a lack of long-term survival data (42). The authors also evaluated loading with oxaliplatin-eluting in D-TACE; the 6 months ORR and DCR were 54.5 and 63.3%, respectively. Thus, its higher risk of allergic reactions and neurotoxicity may limit broad applicability compared to anthracyclines (24). Another study attempted to use target drugs, callispheres beads loaded with donafenib (DCBs). The study found that DCB administration via the hepatic artery was an effective and safe treatment for a preclinical liver cancer model (43). In this study, we used epirubicin-loaded microspheres, the ORR and DCR showed high efficacy. Epirubicin remains a widely recommended option per EASL guidelines, whereas newer drugs lack consensus support (4). However, the quality of life outcomes and functional status changes were valuable, more perspectives and well-designed studies are still needed.

This study enrolled 62 patients with large HCC, all of whom underwent at least one session of D-TACE followed by D-RFA, achieving a technical success rate of 100%. A total of 143 lesions were treated. At one-month follow-up, liver function parameters showed no statistically significant differences compared to baseline (P > 0.05). Notably, no procedure-related mortality (e.g., tumor rupture or liver failure) occurred within the first week. The majority of complications were moderate. One patient experienced rib artery injury following the ablation of a liver tumor located near the right lower lung (segment VII), leading to moderate hemothorax. The patient recovered following aggressive management, including fluid resuscitation, blood transfusion, and prophylactic antibiotics. Tumor lysis syndrome developed in two patients, manifesting as mild to moderate transient hematuria post-procedure. Immediate management with urinary alkalinization, intravenous fluid resuscitation, and diuretics achieved complete resolution of hematuria within 24 h, without other significant adverse effects. Postoperative intermittent fever was noted in many patients, lasting 3 to 5 days. Some patients experienced mild appetite loss or dull abdominal pain. Those with fever exceeding 39°C and accompanying chills received antipyretic treatment. These systemic reactions were consistent with post-embolization syndrome, secondary to extensive tumor necrosis-induced aseptic inflammation following the procedure. The analysis confirmed that the D-TACE followed by D-RFA treatment modality is both safe and stable for unresectable large HCC. The findings align with previous literature, indicating that combining TACE with thermal ablation does not significantly increase the incidence of severe complications (44). For patients with tumors >10 cm and undergoing more than two treatment cycles, the economic burden of medical treatment increased correspondingly. Conversely, for tumors measuring 5 to 7 cm achieving CR after single-cycle therapy, total treatment costs were comparable to or lower than conventional therapies, with superior clinical outcomes. In summary, the sequential D-TACE/D-RFA regimen represents an effective therapeutic strategy for large HCC, with acceptable safety profiles, particularly suitable for surgically ineligible patients.

This study presents several notable advantages in the treatment of large HCC using the D-TACE followed by D-RFA approach. For instance, enhanced power output was possible. The use of two independent RFA machines operating in parallel provided greater energy output compared with traditional systems that utilize a single machine with alternating electrodes. This allows for a single complete ablation of liver tumors exceeding 6 cm, significantly reducing ablation time to as little as 10 minutes, consequently decreasing general anesthesia duration and enhancing hemodynamic stability during the procedure. Improved ablation conformity: The technique offers better ablation conformance, enabling the creation of dual or multiple needle arrangements tailored to the tumor size, shape, and proximity to critical surrounding organs. CT imaging guidance is recommended, with a needle spacing of 2 to 3 cm. This approach facilitates more effective coverage of the planning target volume (PTV), resulting in thorough ablation and reduced recurrence rates. Maximized therapeutic effects were achieved by the sequential application of D-RFA after D-TACE within 1–4 weeks, which allowed to optimize the effects of intra-arterial chemotherapy and embolization. Haiyan et al. analyzed the effects of time interval of ablation treatment after TACE. They found that sequential ablation treatment within 4 weeks after TACE was more effective than delayed ablation, the same was observed in the subgroup analysis in patients with tumor size >5 cm (45). Some studies have reported that the degree of liver function injury in terms of AST, ALT, and TBIL levels, was more serious in DEB-TACE group at 1 week, but was returned to baseline in 4 weeks (46, 47). Therefore, rigorous liver function testing is required within 4 weeks. This strategy effectively reduces heat sink effects during ablation and expands the area of complete tumor necrosis. Stringent quality control is necessary. Preoperative quality control and intraoperative protocol optimization should be rigorously implemented. Ablation range and intensity should be adjusted based on the patient’s physical condition and tumor volume. Postoperative assessment results may help guide subsequent treatment modalities and cycles, contributing to improved efficacy and a reduction in severe complications. Enhanced postoperative care consisting of strict postoperative nursing protocols should be employed to monitor patients and prevent complications such as nausea, allergies, thrombosis, and tumor lysis syndrome. These measures significantly decrease the adverse effects and complications associated with treatment. Effective pain management, such as the use of patient-controlled analgesia (PCA) during the perioperative period has proven effective, easy to operate and highly safe. This approach markedly enhances patient tolerance to the procedure and encourages active participation throughout the treatment process. Overall, the D-TACE followed by D-RFA treatment strategy for large HCC demonstrates significant advantages, including enhanced safety, efficacy, and patient comfort, establishing it as a promising option for managing this challenging condition.

This study has several limitations. First, this was a single-center, retrospective, single-arm study with a small sample size, which is likely to have led to selection bias and does not provide a comparator for the experimental therapy. Most the patients received combination with systemic therapy, the subgroup analyses between those who did and did not may have led to underpowered conclusions. Prospective studies with a larger population from multiple centers are needed to verify the results. Second, differences in the degree of superselective embolization and tumor heterogeneity can result in inconsistent efficacy of D-TACE treatments. Although C-TACE is well-established, our focus on D-TACE was a novel approach. In order to enhance reproducibility, we described the operational process in detail. Third, increased economic burden deriving from the sequential D-TACE and D-RFA approach may lead to higher healthcare costs after multiple treatment cycles. There is hope that large-scale procurement of consumables at reduced prices could alleviate this financial burden. Fourth, the optimal interval times and number of treatment cycles for the new combined modality are not yet established. This lack of standardized guidelines can hinder the ability to replicate results and generalize findings across different patient populations. Despite its promising outcomes, this study acknowledges the aforementioned limitations, which highlight the need for further research to address these challenges and optimize the D-TACE followed by D-RFA treatment protocol for large HCC.

## 5 Conclusion

The combined application of D-TACE and sequential D-RFA for unresectable large HCC has shown clinically significant advantages, including procedural safety, consistent device performance, and effective local tumor control. Furthermore, postoperative adverse events were generally mild, and the combined therapy demonstrated good tolerability across age groups, enabling repeated treatment cycles. This therapeutic strategy significantly improved medium- and long-term survival rates while maintaining quality of life, establishing D-TACE and sequential D-RFA as a viable clinical option. However, patients with exceptionally large tumors may experience increased hospitalization costs. Large-scale multicenter randomized controlled trials are warranted to validate the long-term oncological outcomes.

## Data Availability

The original contributions presented in the study are included in the article/supplementary material. Further inquiries can be directed to the corresponding author.
